# Comments on “Tuberculosis infection control in MDR-TB designated hospitals in Jiangsu Province, China”

**DOI:** 10.1016/j.jctube.2026.100581

**Published:** 2026-01-07

**Authors:** Sushma Narsing Katkuri, Varshini Vadhithala, Arun Kumar, Sushma Verma, Dhanya Dedeepya

**Affiliations:** a Department of Community Medicine, Malla Reddy Instiute of Medical Sciences, Malla Reddy Vishwavidyapeeth, Suraram, Hyderabad 500055 Telangana, India; b Dr. D. Y. Patil Medical College, Hospital and Research Centre, Dr. D. Y. Patil Vidyapeeth (Deemed-to-be-University), Pimpri, Pune 411018 Maharashtra, India; c Faculty of Pharmaceutical Sciences, Graphic Era Hill University, Dehradun, India; d Centre for Promotion of Research, Graphic Era Deemed University, Dehradun, India; e Department of Pharmaceutics, Noida Institute of Engineering & Technology (Pharmacy Institute), Plot No.19, Knowledge Park-II, Greater Noida, India; f Saveetha Medical College and Hospital, Saveetha Institute of Medical and Technical Sciences, Saveetha University, Chennai, India; g Department of Anatomy, School of Medical Sciences and Research. Sharda University, Greater Noida, India

**Keywords:** Tuberculosis infection control, TB designated hospitals, TB prevention, High TB burden countries

Dear editor,

Song et al. present a valuable multicentre before–after evaluation of tuberculosis infection control (TBIC) in MDR-TB–designated hospitals in Jiangsu Province, China, reporting substantial improvements in administrative, environmental and respiratory protection measures over time [1]. The alignment of their intervention package with WHO’s hierarchical TB infection prevention and control framework and use of a standardized assessment tool represent important strengths [2].

Several methodological aspects, however, warrant clarification to help readers interpret the magnitude and robustness of the reported effects. First, there is an internal inconsistency in the study timeline. The abstract states that baseline evaluation was completed by January 2019 with more than two years of follow-up concluding on 31 August 2021, whereas the Methods describe a before–after study “from October 2019 to September 2021.” These statements cannot simultaneously be correct and leave uncertainty about the true observation window and the number of quarterly assessments per site. Because before–after designs are sensitive to secular trends and time-varying confounders, precise specification of the study period is critical for valid inference [3].

Second, the design is described as a before–after study across six hospitals, without a concurrent control group. Quasi-experimental pre–post designs are widely used in infection-control research, but they are prone to overestimating intervention effects when background changes (e.g. COVID-19–related infection-prevention culture, policy shifts, or resource injections) coincide with implementation [4]. Even simple strategies such as interrupted time-series analysis, or inclusion of suitable comparison units where feasible, can substantially strengthen causal interpretation in such settings [3], [4].

Third, the primary outcomes are composite implementation scores constructed from ordinal items (0/1/2) for each indicator, aggregated to hospital and department level and then analysed as continuous means. While this is common practice in quality-improvement work, it assumes equal weighting and linearity across items and does not describe how heterogeneity in the number of applicable indicators between sites was handled. Without clarity on weighting and on whether hospitals with more indicators contribute disproportionately to overall means, comparisons across facilities and over time may be difficult to interpret.

Fourth, the analysis appears to ignore clustering. Only six hospitals were included, yet inferential statistics (p-values) are reported for changes in mean implementation rates. With such a small number of clusters and repeated measures within each, conventional parametric tests that treat observations as independent will underestimate uncertainty. More appropriate approaches would include hospital-level analyses, presentation of absolute changes with confidence intervals, or mixed-effects models with explicit recognition of the hierarchical structure, while accepting the limited power inherent in the design [3].

Finally, assessment teams included local CDC staff and hospital personnel involved in TBIC, and were not blinded to the intervention phase. This, together with repeated use of the same tool, may have introduced measurement drift and optimism bias, particularly for items dependent on subjective judgement. Explicit discussion of these potential biases would further contextualise the large observed improvements.

These considerations do not detract from the operational importance of Song et al.’s work but suggest that the magnitude and generalisability of the reported effects should be interpreted with caution. Clarification of the study timeline, analytic methods and handling of clustering would strengthen confidence in the conclusions and provide a useful methodological model for future TBIC evaluations.

## Declaration of competing interest

The authors declare that they have no known competing financial interests or personal relationships that could have appeared to influence the work reported in this paper.
